# Ultrasound Muscle Thickness Is a Poor Index of Criterion Magnetic Resonance Imaging Measures of Resistance Training-Induced Muscle Growth

**DOI:** 10.1249/MSS.0000000000004078

**Published:** 2026-07-07

**Authors:** THOMAS G. BALSHAW, MARK P. FUNNELL, EMMET J. MCDERMOTT, THOMAS M. MADEN-WILKINSON, LEWIS J. JAMES, JONATHAN P. FOLLAND

**Affiliations:** 1 National Centre for Sport & Exercise Medicine, School of Sport, Exercise, and Health Sciences, Loughborough University, Leicestershire, UNITED KINGDOM; 2 National Institute for Health and Care Research (NIHR) Leicester Biomedical Research Centre, University Hospitals of Leicester National Health Service (NHS) Trust and the University of Leicester, Leicester, Leicestershire, UNITED KINGDOM; 3 NIHR Applied Research Collaboration East Midlands Diabetes Research Centre, University of Leicester, Leicester, Leicestershire, UNITED KINGDOM; 4 Department of Physical Education and Sport Sciences, University of Limerick, Limerick, IRELAND; 5 Emles Bioventures, Wimbledon, London, UNITED KINGDOM

**Keywords:** KNEE EXTENSORS, MUSCLE HYPERTROPHY, MUSCULOSKELETAL IMAGING, STRENGTH TRAINING

## Abstract

**Introduction:**

The purpose of this study was to compare group-level resistance training (RT)-induced changes in ultrasound-measured muscle thickness (ΔMT_US_) with criterion magnetic resonance imaging (MRI) measures of muscle growth (i.e., changes in maximum anatomical cross-sectional area [ΔACSA_MRI_] and volume [ΔVOL_MRI_]), and examine the relationship between ΔMT_US_ and both ΔACSA_MRI_ and ΔVOL_MRI_, for the quadriceps femoris (QF) and its constituent muscles.

**Materials and Methods:**

Healthy young males (*n* = 39) completed 15 weeks of lower-body RT (knee extension, knee flexion, and leg press; ×3 sessions/wk). Pre- and post-RT participants undertook dominant thigh musculoskeletal scans. Ultrasound and MRI scans were manually segmented to quantify pre-to-post ΔMT_US_, ΔACSA_MRI_, and ΔVOL_MRI_ of constituent muscles and the overall QF.

**Results:**

ΔMT_US_ significantly underestimated increases in ΔACSA_MRI_ (0.001 ≤ *P* ≤ 0.002) and ΔVOL_MRI_ (0.001 ≤ *P* ≤ 0.012) for the overall QF and two out of four constituent muscles (vastus lateralis and vastus medialis), with a mean offset/underestimate of more than half the change in criterion MRI measures, and an average individual error that was typically more than the change in criterion MRI measures. Although vastus intermedius and rectus femoris ΔMT_US_ did not differ compared with ΔACSA_MRI_, and ΔVOL_MRI_ (0.057 ≤ *P* ≤ 0.113). There were no significant bivariate correlations between ΔMT_US_ and either ΔACSA_MRI_ or ΔVOL_MRI_ for the overall QF (−0.152 ≤ r ≤ −0.015, 0.393 ≤ adjusted *P* ≤1.431) or any constituent muscles of the QF (−0.197 ≤ r ≤ 0.422, 0.140 ≤ adjusted *P* ≤1.002) in absolute or relative terms.

**Conclusions:**

Overall, ΔMT_US_ was not an accurate or valid index of RT-induced muscle growth, and it is recommended to exercise substantial caution in the use and interpretation of MT_US_ in the context of RT adaptation.

Skeletal muscle generates forces that are transmitted, via tendinous tissue, to the skeleton, facilitating movement. Skeletal muscle size is a primary determinant of maximum strength (1–4), with losses of muscle mass because of mechanical unloading following injury (5), musculoskeletal disease (6), or sarcopenia (7) contributing to reductions in maximum strength. Regular resistance training (RT) is a potent stimulus for increasing muscle mass that is widely recommended for all adults (8,9), and recent evidence suggests that muscle growth is the key driver of the individual changes in strength after RT (4). Consequently, much research attention has been dedicated to optimizing RT prescription (10,11) and accompanying nutritional supplementation (12–14) for maximizing skeletal muscle growth.

High-resolution magnetic resonance imaging (MRI) is widely considered the criterion method for assessing muscle size, and thus also changes in muscle size following RT (15). However, MRI scanning is expensive, time-intensive, requires specialist expertise, and is not widely accessible (16). Consequently, researchers often employ alternate approaches to measure RT-induced muscle growth. Muscle thickness acquired via B-mode ultrasound scanning (MT_US_) is a unidimensional measurement of muscle size, measured as the distance between the superficial and deep aponeuroses (1), that provides a relatively low-cost, and portable alternative to MRI scanning that also permits rapid image acquisition and analysis.

Thus far most research attention has focused on the accuracy of MT_US_ to assess muscle size per se (17–23), and studies have typically reported moderate to strong relationships between MT_US_ compared with MRI measures in these cross-sectional analyses (0.469 ≤ r ≤ 0.922). However, MT_US_ has also been widely used to assess RT-induced muscle growth, that is, changes in MT_US_ (ΔMT_US_; (24–31)). Yet, little attention has been given to the validity of ΔMT_US_ compared with criterion measures of muscle growth with RT, that is, MRI-derived changes in maximum anatomical cross-sectional area (ΔACSA_MRI_) or volume (ΔVOL_MRI_). Given that the changes in muscle size with RT are of a much smaller magnitude (~5–15%) of cross-sectional measures of muscle size per se, measurement precision may be more important in the context of assessing muscle growth (i.e., change) after RT.

To our knowledge, only 1 study has compared group-level responses between ΔMT_US_ and ΔACSA_MRI_ after a period of RT (32), and only two studies have investigated if individual ΔMT_US_ are related to ΔACSA_MRI_ after RT (20,33). After 6 weeks of RT in a small sample of young men (*n* = 10), group-level ΔMT_US_ of the elbow flexors did not consistently equate ΔACSA_MRI_ (32). Additionally, using small samples for the assessment of relationship, individual participant ΔMT_US_ of the vastus lateralis (VL) following 10–12 weeks of RT has been reported to be related (r = 0.69; *P* = 0.001; *n* = 9; (20)) and unrelated (r = 0.539; *P* = 0.071; *n* = 12; (33)) to the ΔACSA_MRI_ of the same muscle. Curiously, in the VL study where ΔMT_US_ was reported to be related to ΔACSA_MRI_, ΔMT_US_ was not related to ΔVOL_MRI_ (r = 0.33; *P* = 0.207; (20)). Therefore, it is currently unclear if ΔMT_US_ reflects MRI measures of muscle growth, and no studies have assessed the validity of ΔMT_US_ after RT for muscles other than the VL (e.g., the other constituent muscles of the quadriceps femoris [QF]) or indeed a whole muscle group such as the QF. Assessing the size of whole muscle groups is the conventional, more comprehensive, and functionally relevant approach for assessing RT adaptations (14,34–37).

Moreover, ΔMT_US_ and changes in MRI measures of ACSA_MRI_ and VOL_MRI_ are inherently different in terms of both imaging modality and geometry. While MRI is often considered to offer better soft tissue contrast resolution, for some applications ultrasound has been found to offer equivalent (38) or better (39) accuracy. From a geometric perspective, assuming a simplified muscle with a circular cross-section undergoing purely radial growth, MT increases in proportion with the linear dimension (i.e., radius, r), whereas ACSA increases to the square of the linear dimension (i.e., r^2^). Hence, in this simplified example, the relative (percentage) changes in MT_US_ might be expected to underestimate those of ACSA, although clearly many skeletal muscles are not circular in cross-section and regional hypertrophy could lead to nonuniform changes in shape (40–42).

Accordingly, the purpose of the present investigation was to compare group-level RT-induced ΔMT_US_ with ΔACSA_MRI_ and ΔVOL_MRI_, and examine the relationship between ΔMT_US_ and changes in MRI assessments of individual participants following RT, for the QF and its constituent muscles. It was hypothesized that group-level ΔMT_US_ would underestimate those observed with MRI-based measures and that ΔMT_US_ would demonstrate significant bivariate correlations with muscle growth assessed with ACSA_MRI_ for the QF overall and each constituent muscle.

## MATERIALS AND METHODS

### Participants

Fifty-two healthy, recreationally active young men were recruited to the study, of whom 39 completed the intervention period (age, 26 ± 4 years; height, 1.79 ± 0.08 m; body mass, 74.6 ± 11.7 kg; habitual physical activity, assessed via the short format International Physical Activity Questionnaire (43), 2145 ± 1447 MET-min/wk). Thirteen participants withdrew during the study (weeks 1–5, *n* = 4 dropouts; weeks 6–10, *n* = 5 dropouts; weeks 11–15, *n* = 4 dropouts) for the following reasons: changes in personal circumstances (×4); low enjoyment of RT regime (×3); nonattendance (×2); or knee discomfort (×4). All participants provided their written informed consent to participate in this study and had no recent history of RT (for over 18 months), as well as low to moderate habitual physical activity levels. Before participant recruitment, ethical approval was granted by the Loughborough University Ethics Review Sub-Committee (Reference Number: R19-P030). At the point of study admission, participants were screened for protein intake, via 24 h food intake recall, and excluded if they had habitual protein intake <0.8 g/kg.

### Overview

The data used in this study were taken from a previously published report produced by our laboratory that investigated the effects of a bioactive collagen peptide supplement on muscle growth compared with a placebo supplement following 15 weeks of lower-body RT (14). Given that both groups within this study experienced pre-to-post increases in all constituent QF muscles (as quantified by ΔVOL_MRI_), the two groups were collapsed to produce a total cohort of *n* = 39. Ultrasound and MRI scans of the dominant thigh (as determined from leg preference for kicking a ball) were conducted pre- (5–7 days before the first training session) and post-RT (3–5 days after the final training session). Scan time of day was standardized within participants, who were instructed to eat and drink normally and to avoid strenuous exercise and alcohol consumption for 36 h before all measurement sessions. During the 15-week intervention, all participants completed a lower-body RT program three times per week (45 sessions in total) that primarily targeted the QF. Participants were instructed to maintain their habitual physical activity and usual diet throughout the study.

### Resistance Training

All participants completed the same supervised, undulating periodized (~12 to ~6 repetition maximum), progressive lower-body RT program 3 times per week (typically Monday, Wednesday, Friday) during the 15-week intervention period, as described elsewhere (14). Briefly, each session was fully supervised by the investigators and involved three isoinertial (constant load) exercises (2–4 sets of each exercise and 4 sets from week 7 onwards), in the following order: unilateral knee extension (TechnoGym, Selection Leg Extension; Bracknell, UK) commencing with the dominant leg and alternating sets between legs; bilateral knee flexion (Life Fitness, Seated Leg Curl SL40; Cambridgeshire, UK); bilateral leg press (Watson Gym Equipment, 45° Leg Press, Somerset, UK). Two minutes of recovery separated sets with the same leg/legs. Participants took 2 s to lift and 2 s to lower the weight during each exercise. Progressive overload was achieved by increasing load if all specified repetitions in the penultimate set of an exercise were completed. Bilateral warm-up sets were completed before unilateral knee extension and bilateral knee flexion (same number of repetitions at ~50% of the load for the first main set).

### Ultrasonography

MT_US_ of each constituent QF muscle (vastus medialis [VM], VL, vastus intermedius [VI], and rectus femoris [RF]) was measured using B-mode ultrasonography (EUB-8500, Hitachi Medical Systems UK Ltd, Northamptonshire, UK) using a 92 mm long, 10 MHz linear-array transducer (EUP-L53L) coated with water-soluble transmission gel (Figure 1 A, Supplemental Digital Content, available at https://links.lww.com/MSS/D445). Specifically, 2–3 MT measurements were made at different locations along each constituent muscle for a total of 11 MT measurements across the quadriceps. Participants sat at rest for 10–15 min in an isometric dynamometer (knee and hip angles of 101° and 126°, respectively) while ultrasound imaging locations were marked. Ultrasound image recording sites were marked on the skin at specific percentages of thigh length above the knee joint space: 50% (VI and VL); 20% (VM); 75% (RF) and in all cases at 50% of superficial medio-lateral muscle width. These imaging locations were selected such that the longitudinal image spanned the expected site of ACSA_MRI_, based on our previous MRI observations of muscle-specific ACSA-femur length curves (1,44).

The center of the 92 mm ultrasound transducer was positioned at the specified location and parallel to the thigh’s long axis and perpendicular to the skin, so that the deep and superficial aponeuroses were clearly identifiable with minimal pressure applied to the dermal surface. Ultrasound video output was recorded by a PC (via an S-video to USB video converter [VCAP302, ClimaxDigital, Spennymoor, UK] and Spike video recorder software [CED Ltd., Cambridge, UK]). Images were analyzed off-line by one trained investigator using public domain software (Image J, v1.48, National Institutes of Health, Bethesda, USA). MT was measured as the distance between the superficial and deep aponeurosis at three points within each image: the left (−36 mm, distal end), center (0 mm), and right (+36 mm, proximal end) of the image (RF, VI, and VL). Except for the VM, in which case MT was measured at two points within each ultrasound image (center [0 mm], and right [+36 mm, proximal]) to closely equate the location where the peak of the ACSA-femur length curve occurs (i.e., ~25%–30% of femur length, 0% = distal; (1,44)). These multiple measures of thickness for each muscle were then averaged to produce a final MT_US_ value for the given constituent muscle. Overall QF MT_US_ was derived as the sum of the four constituent muscles. Images (ultrasound and MRI) were analyzed blind for all other values, but not for the measurement time point (pre/post).

### MRI Scan and Analysis

Participants sat quietly for 15 min before MRI scans (3.0 T Discovery MR750w, GE Healthcare, Chicago, IL). T1-weighted axial and coronal plane MRI scans of the dominant thigh (between the anterior superior iliac spine to the lateral tibial condyle) were conducted with participants supine, arms resting across the chest, hip and knee joints extended, and the ankle at ~90°. Using a receiver 8-channel whole-body coil, axial (time of repetition/time to echo 600/8.35 ms; image matrix 512 × 512; field of view 260 mm × 260 mm; pixel size 0.508 mm × 0.508 mm; slice thickness 5 mm; inter-slice gap 0 mm) and coronal (time of repetition/time to echo 600/8.53 ms; image matrix 256 × 256, field of view 450 mm × 450 mm, pixel size 1.76 mm × 1.76 mm, slice thickness 5 mm, inter-slice gap 0 mm) images were acquired in two overlapping blocks for both axial and coronal scans. Synchronization of coronal and axial plane scans permitted the objective alignment of axial blocks during analysis (Horos, version 3.3.6, https://horosproject.org/).

To determine VOL_MRI_ of constituent muscles (RF, VL, VM, and VI), they were segmented in every third image (i.e., every 15 mm) along each muscle, from the first distal image in which that muscle appeared until the muscle was no longer visible (Figure 1 B, Supplemental Digital Content, available at https://links.lww.com/MSS/D445). The following number of images were segmented along the length of each muscle (mean of pre- and post-image number before calculating a mean across all participants): RF, 27 (range 24–30); VM, 27 (range 24–30); VI, 29 (range 27–33); VL, 29 (range 25–31). The VOL_MRI_ of each muscle was calculated as the area under the ACSA-muscle length curve following cubic spline interpolation (1000 points; GraphPad Prism 8; GraphPad Software). ACSA_MRI_ was measured as the maximum ACSA of all the imaged slices of each muscle. Based on the initial measurements in every third slice (for VOL_MRI_ described above) and visual inspection, seven contiguous slices around the suspected maximum ACSA (i.e., 3 either side) were assessed. This ensured that the single image with the highest ACSA of each constituent muscle was measured. Constituent muscle VOL_MRI_ or ACSA_MRI_ were summed to determine overall QF measures.

### Reliability

A pilot trial of nine healthy young men involved two identical sessions of musculoskeletal imaging (both ultrasound and MRI in each session, with sessions 5–14 days apart), using the aforementioned protocols and analysis procedures, was used to establish within-participant reliability (i.e., within-participant coefficient of variation, CV_W_; [SD/mean] × 100) of MT_US_ measures and MRI-derived ACSA_MRI_ and VOL_MRI_). Within-participant between-test session reliability of MT_US_ measures was determined to be 2.9 ± 2.6%, 3.4 ± 1.8%, 4.2 ± 5.9%, 2.1 ± 1.7%, and 1.6 ± 1.9% for the VL, VI, VM, RF, and QF, respectively. Within-participant between-test session reliability of ACSA_MRI_ was 1.1 ± 0.9%, 2.3 ± 1.8%, 1.1 ± 0.9%, 0.6 ± 0.5%, and 1.1 ± 0.7%, for the VL, VI, VM, RF, and QF, respectively. Within-participant between-test session reliability of VOL_MRI_ was 1.5 ± 1.4%, 1.5 ± 0.7%, 1.2 ± 0.7%, 1.3 ± 1.3%, and 0.9 ± 0.9%, for the VL, VI, VM, RF, and QF, respectively.

### Statistical Analysis

Data normality of absolute pre- and post-test measures and change data (absolute and percentage) was assessed using the Shapiro-Wilk test. Twelve out of 15 absolute pretest measures and 13 out of 15 post-test measures were normally distributed. Where a pre- or post-test measure was not normally distributed, absolute pre- to postdata comparisons were completed using Wilcoxon matched pairs signed rank tests; otherwise, group-level changes from pre-to-post in the measures of muscle size (MT_US_, ACSA_MRI_, and VOL_MRI_) were assessed with paired *t* tests. Thirteen out of 15 absolute change measures and 14 out of 15 percentage change measures were normally distributed. When comparing group-level percentage change between measures (e.g., VM MT_US_ vs VM ACSA_MRI_ vs VM VOL_MRI_) comparisons featuring not normally distributed measures were made using Friedman’s test with Dunn’s post hoc testing if necessary to determine where differences occurred. Otherwise, group-level percentage differences between corresponding measures were assessed using a one-way analysis of variance (ANOVA) with post hoc comparisons between measures, Bonferroni corrected to account for multiple comparisons to determine where differences occurred. Offset was calculated by subtracting percentage change scores in MRI measures from MT_US_ measures for each participant (i.e., ΔMT_US_ − ΔACSA_MRI_ or ΔVOL_MRI_) and deriving a mean. Root mean square (RMS) error was calculated by: 1) calculating the square of the offset for each participant (ΔMT_US_ − ΔACSA_MRI_ or ΔVOL_MRI_); 2) summing the squared offset values across participants; 3) dividing the sum value by the number of participants; and 4) finally, deriving the square root of the value produced from step (3).

Considering the relationships between individual ΔMT_US_ and ΔACSA_MRI_ or ΔVOL_MRI_, the extra sum of squares *F* test was conducted to compare the goodness-of-fit of two alternative nested models (i.e., linear vs quadratic). All *F*-tests were nonsignificant (0.064 ≤ *F* ≤ 3.714, 0.062 ≤ *P* ≤0.795), indicating the more complex quadratic model was not superior to the simpler linear model for assessing the relationship between ΔMT_US_ and ΔACSA_MRI_ or ΔVOL_MRI_. Hence, linear association was assessed with Pearson’s product-moment correlation. When absolute or percentage change data were not normally distributed, Yeo-Johnson transformations, which accommodate both positive and negative observations, were made. Following transformation, normality of change data was reassessed and confirmed before proceeding with Pearson’s product-moment correlations. One change variable, percentage VI ΔACSA_MRI_, was not normally distributed after transformation, and as a result, a Spearman’s *r* correlation was used to assess the strength of this variable’s relationship with percentage VI ΔMT_US_. Data transformation was performed using RStudio (Version 2025.05.1 + 513, Posit Software, PBC). Pearson’s product-moment correlations were conducted between both absolute and percentage pre- to post-RT: 1) ΔMT_US_ and 2) MRI measures (ΔVOL_MRI_ and ΔACSA_MRI_) for each constituent muscle of the QF and overall measures of the QF (e.g., absolute change: VM ΔMT_US_ [mm] vs VM ΔVOL_MRI_ [cm^3^]; percentage change: VM ΔMT_US_ [%] vs VM ΔVOL_MRI_ [%]). Correlation coefficients were classified as follows: “very weak” (r ≤ 0.30), “weak” (r = 0.30–0.49), “moderate” (r = 0.50–0.69) and “strong” (r = 0.70–0.90). Statistical significance for all statistical tests was defined as *P* < 0.05, and all statistical analysis was completed using GraphPad Prism 10 (GraphPad Software). To correct for the number of bivariate correlations conducted (*n* = 20), the Benjamini-Hochberg procedure with a false discovery rate of 5% was employed to determine the significance of bivariate correlations (45). To retain an alpha value threshold of *P* < 0.05, each *P* value produced from the Pearson’s product-moment correlation was multiplied by the number of statistical tests (*n* = 20) divided by the *P* value rank (i.e., rank of 1 = lowest *P* value; (46) to produce an adjusted *P* value. All data are presented as mean ± standard deviation, unless otherwise indicated.

## RESULTS

### Absolute Group Level Changes

After the 15-week RT intervention, both MRI measures of muscle size increased for each constituent muscle and the overall QF (paired *t* test or Wilcoxon signed rank test, [all] *P* ≤ 0.001; Table 1). Pre-to-post increases in MT_US_ were observed for the overall QF and three out of four constituent muscles (paired *t* test or Wilcoxon signed rank test, 0.001 ≤ *P* ≤ 0.007; Table 1), but not the VM (Wilcoxon signed rank test, *P* = 0.457).

**TABLE 1.** Group level changes in ultrasound muscle thickness (MT_US_) or magnetic resonance imaging (MRI) measures of muscle size (maximum anatomical cross-sectional area [ACSA_MRI_] and volume [VOL_MRI_]) following 15 weeks of lower-body resistance training (*n* = 39).

	MT_US_ (mm)		ACSA_MRI_ (cm^2^)		VOL_MRI_ (cm^3^)	
	Pre	Post	Pre	Post	Pre	Post
VL	26.3 ± 4.5	27.2 ± 4.3^**^	31.4 ± 5.3	35.6 ± 5.4^***^	637 ± 128	730 ± 142^***^
VI	18.3 ± 3.2	19.4 ± 3.3^**^	28.3 ± 5.1	30.7 ± 4.7^***^	603 ± 126	660 ± 116^***^
VM	32.1 ± 4.8	32.7 ± 4.2	25.6 ± 5.0	28.9 ± 4.7^***^	483 ± 100	537 ± 96^***^
RF	22.9 ± 2.3	24.5 ± 2.8^***^	13.7 ± 2.7	15.0 ± 3.1^***^	266 ± 62	297 ± 71^***^
QF	99.5 ± 9.8	103.8 ± 9.5^***^	99.0 ± 15.6	110.1 ± 15.2^***^	1989 ± 381	2224 ± 383^***^

Pre-to-post increases in muscle size determined via paired *t* test or Wilcoxon signed rank test are denoted as follows:

** *P* < 0.01,

*** *P* < 0.001. QF measures are derived as the sum of the constituent muscles (i.e., VL, VI, VM, and RF).

### Percentage Changes: Group-Level Comparisons, Offset and RMS Error

Percentage changes differed between the three muscle size measures for the overall QF, VL, and VM (ANOVA, [all] *P* ≤ 0.001) but not the VI or RF (Friedman’s test or ANOVA, 0.057 ≤ *P* ≤ 0.113; Fig. 1). Post hoc tests revealed percentage changes in overall QF, as well as VL and VM, were smaller for ΔMT_US_ (i.e., a significant underestimate) compared with ΔACSA_MRI_ (0.001 ≤ *P* ≤ 0.002) and ΔVOL_MRI_ (0.001 ≤ *P* ≤ 0.012). Consequently, the mean offset for percentage ΔMT_US_ compared with changes in MRI measures for the QF were −7.3% (ΔACSA_MRI_) and −7.8% (ΔVOL_MRI_), or underestimates of more than half the change in the criterion measures (Table 2). Across constituent muscles, the offset between ΔMT_US_ compared with changes in MRI measures ranged from: −2.5 to −10.4% (ΔACSA_MRI_); and −4.1 to −11.0% (ΔVOL_MRI_). RMS error of ΔMT_US_ compared with the changes in criterion MRI measures for the overall QF were 13.2% (ΔACSA_MRI_) and 12.8% (ΔVOL_MRI_); that is, the average error was greater than the change in the criterion measures. For the constituent muscles, RMS error of ΔMT_US_ compared with the changes in criterion MRI measures ranged from: 13.2 to 19.9% (ΔACSA_MRI_); and 12.8 to 19.6% (ΔVOL_MRI_). There were no differences between percentage ΔACSA_MRI_ and ΔVOL_MRI_ for overall QF or VL (post hoc, [both] *P* > 0.999), but percentage increases in ACSA_MRI_ were greater than VOL_MRI_ for the VM (post hoc, *P* = 0.015). Post hoc analysis was not conducted for the RF or VI as percentage changes did not differ between the three measures of muscle size (Friedman’s test or ANOVA, 0.057 ≤ *P* ≤ 0.113).

**TABLE 2.** Difference between resistance training-induced percentage changes (Δ) in: (1) ultrasound muscle thickness (MT_US_) measures and; (2) criterion magnetic resonance imaging (MRI) measures of muscle size (maximum anatomical cross-sectional area [ACSA_MRI_] and volume [VOL_MRI_]) for constituent muscles and the overall quadriceps femoris (QF).

Muscle/Muscle Group	Criterion Measure (%)	*ΔMT*_*US*_ vs. *Δ* Criterion (%)			RMS Error (%)
		Mean Offset	95% Confidence Intervals		
			Lower	Upper	
	ΔACSA_MRI_				
VL	14.4	−10.3	−14.3	−6.3	16.3
VI	9.0	−2.7	−6.8	1.4	13.2
VM	13.5	−10.4	−15.8	−5.0	19.9
RF	10.2	−2.5	−7.0	2.1	14.5
QF	11.9	−7.3	−10.8	−3.8	13.2
	ΔVOL_MRI_				
VL	15.1	−11.0	−14.2	−7.8	14.9
VI	10.5	−4.1	−7.9	−0.3	12.8
VM	11.9	−8.8	−14.4	−3.2	19.6
RF	11.9	−4.2	−8.5	0.0	14.1
QF	12.4	−7.8	−11.0	−4.6	12.8

Offset was calculated by subtracting percentage change (∆) scores in MRI measures from MT_US_ measures, that is, Δ MT_US_ ─ Δ ACSA_MRI_ or VOL_MRI_.

QF measures were calculated by summing the values of the four constituent muscles of the QF.

Negative values indicate that percentage changes in MTUS measures underestimated muscle size changes relative to MRI measures. Positive value indicates that percentage changes in the MT_US_ measure overestimated muscle size changes relative to MRI measures.

**FIGURE 1:**
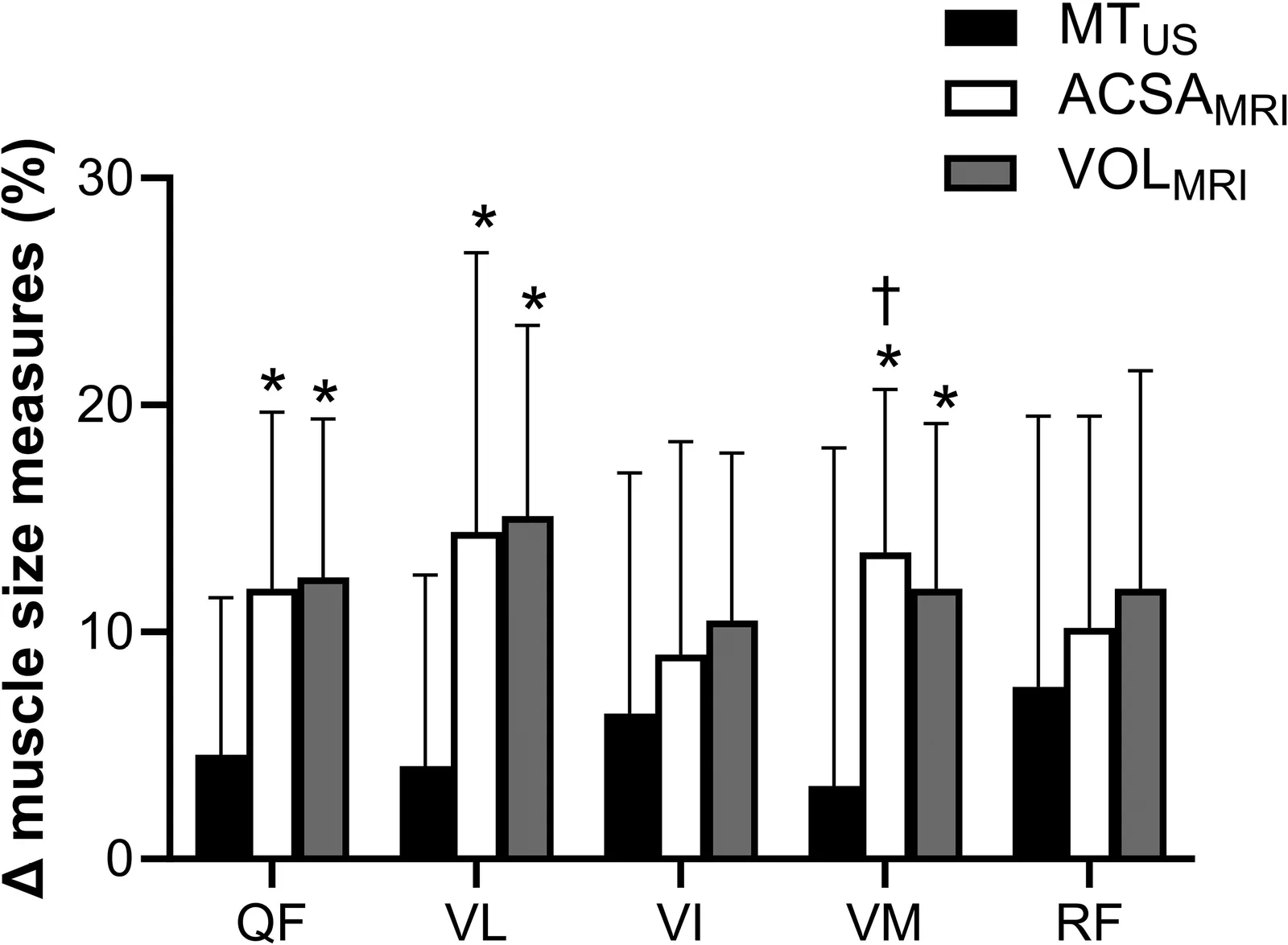
A comparison of percentage changes (Δ) between MT_US_ and magnetic resonance imaging measures of muscle size (ACSA_MRI_ and VOL_MRI_) following 15 weeks of lower-body resistance training (*n* = 39). Values are mean ± SD. *Denotes significantly greater percentage change compared with MT_US_. †denotes significantly greater percentage change compared to VOL_MRI_.

### Bivariate Correlations Between Changes in MT_US_ and MRI measures of muscle size

The absolute ΔMT_US_ and ΔACSA_MRI_ were unrelated for the QF (r = −0.110, adjusted *P* = 0.634; Fig. 2A) or any of the constituent muscles (−0.096 ≤ r ≤ 0.240, 0.490 ≤ adjusted *P* ≤ 0.745; Fig. 2, B–E). Similarly, absolute ΔMT_US_ and ΔVOL_MRI_ were not related for the QF overall (r = −0.015, adjusted *P* = 1.431; Fig. 3A) or any of the constituent muscles (−0.153 ≤ r ≤ 0.422, 0.140 ≤ adjusted *P* ≤ 0.866; Fig. 3, B–E). For relative (percentage) changes, there were also no bivariate correlations between percentage ΔMT_US_ and ΔACSA_MRI_ for the QF overall (r = −0.152, adjusted *P* = 0.393; Fig. 4A) or any of the constituent muscles (−0.117 ≤ r ≤ 0.281, 0.564 ≤ adjusted *P* ≤ 1.002; Fig. 4, B–E). There were no relationships between relative (percent) ΔMT_US_ and ΔVOL_MRI_ for the QF overall (r = −0.091, adjusted *P* = 0.833; Fig. 5A) or any of the constituent muscles (−0.197 ≤ r ≤ 0.281, 0.229 ≤ adjusted *P* ≤ 0.869; Fig. 5, B–E).

**FIGURE 2:**
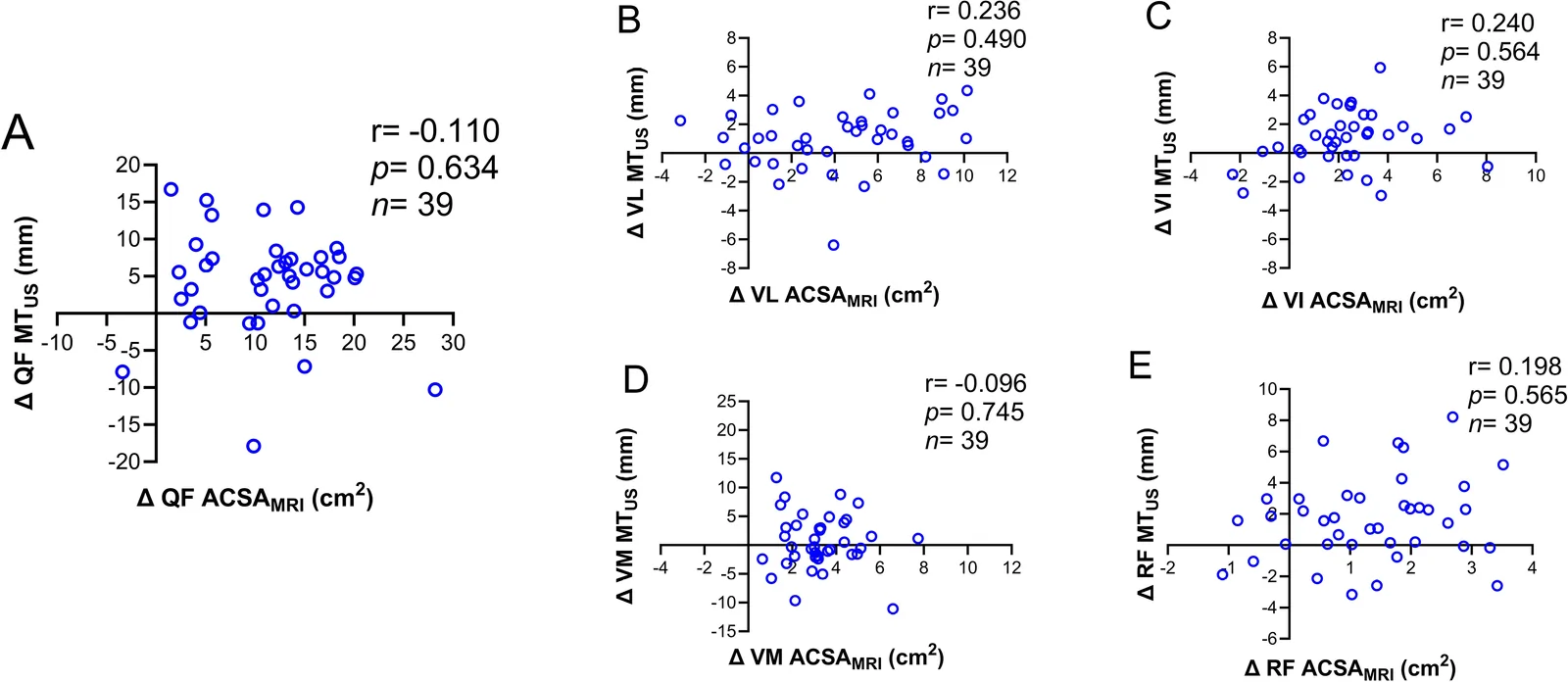
Bivariate correlations between absolute changes (Δ) in MT_US_ and changes in ACSA_MRI_ of the overall quadriceps femoris (QF; A) and the constituent muscles (B–E) following 15 weeks of lower-body resistance training. r values derived from Pearson’s product-moment correlations are displayed. Adjusted *P* values are also displayed.

**FIGURE 3:**
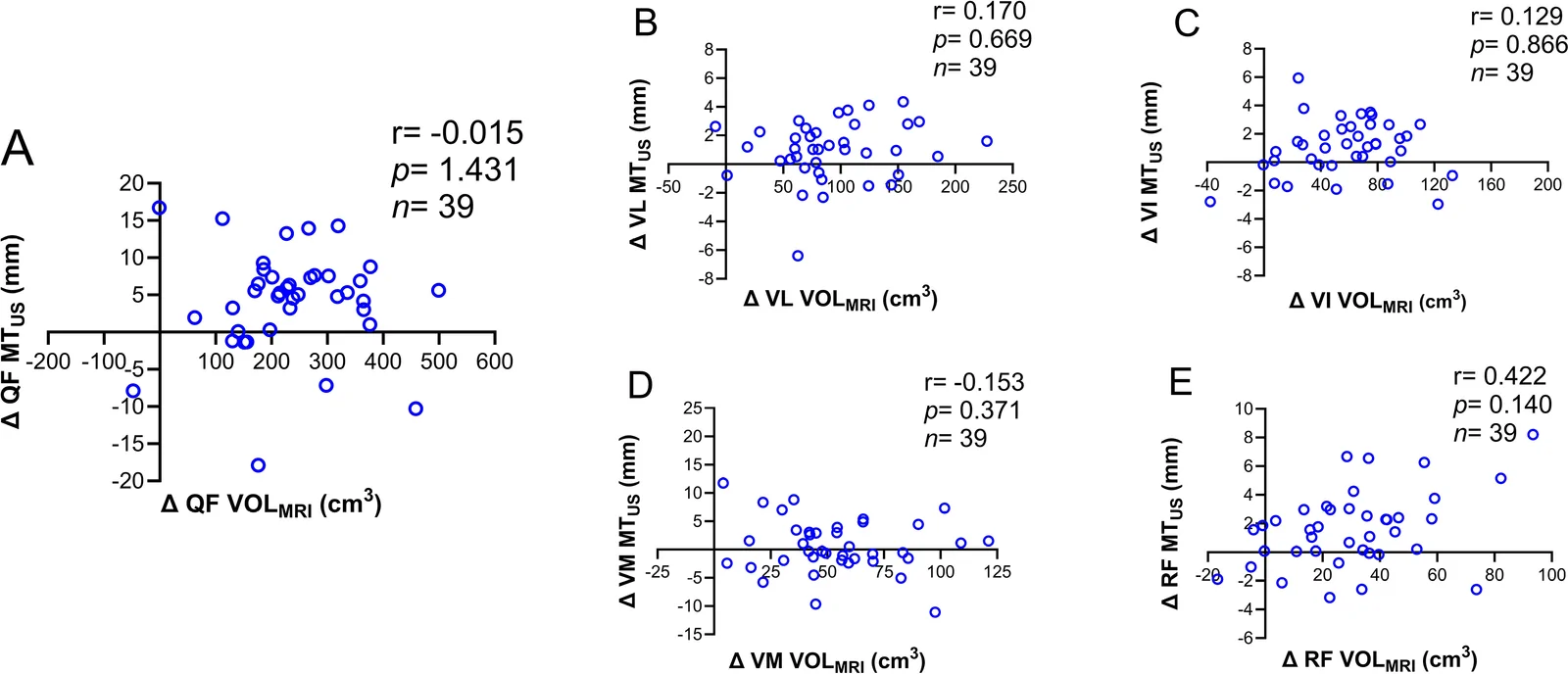
Bivariate correlations between absolute changes (Δ) in MT_US_ and changes in VOL_MRI_ of the overall quadriceps femoris (QF; A) and the constituent muscles (B–E) following 15 weeks of lower-body resistance training. r values derived from Pearson’s product-moment correlations are displayed. Adjusted *P* values are also displayed.

**FIGURE 4:**
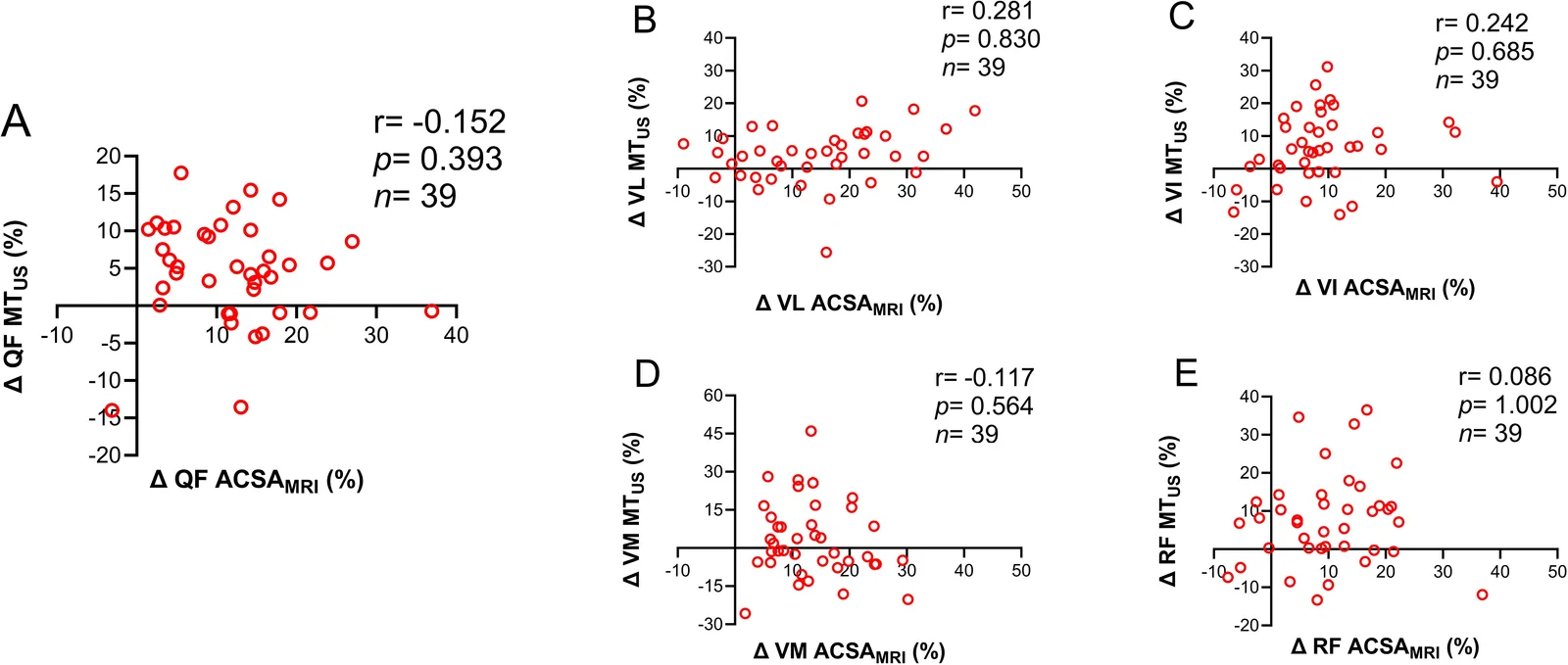
Bivariate correlations between percentage changes (Δ) in MT_US_ and changes in ACSA_MRI_ of the overall quadriceps femoris (QF; A) and the constituent muscles (B–E) following 15 weeks of lower-body resistance training. r values derived from Pearson’s product-moment correlations are displayed. Adjusted *P* values are also displayed.

**FIGURE 5:**
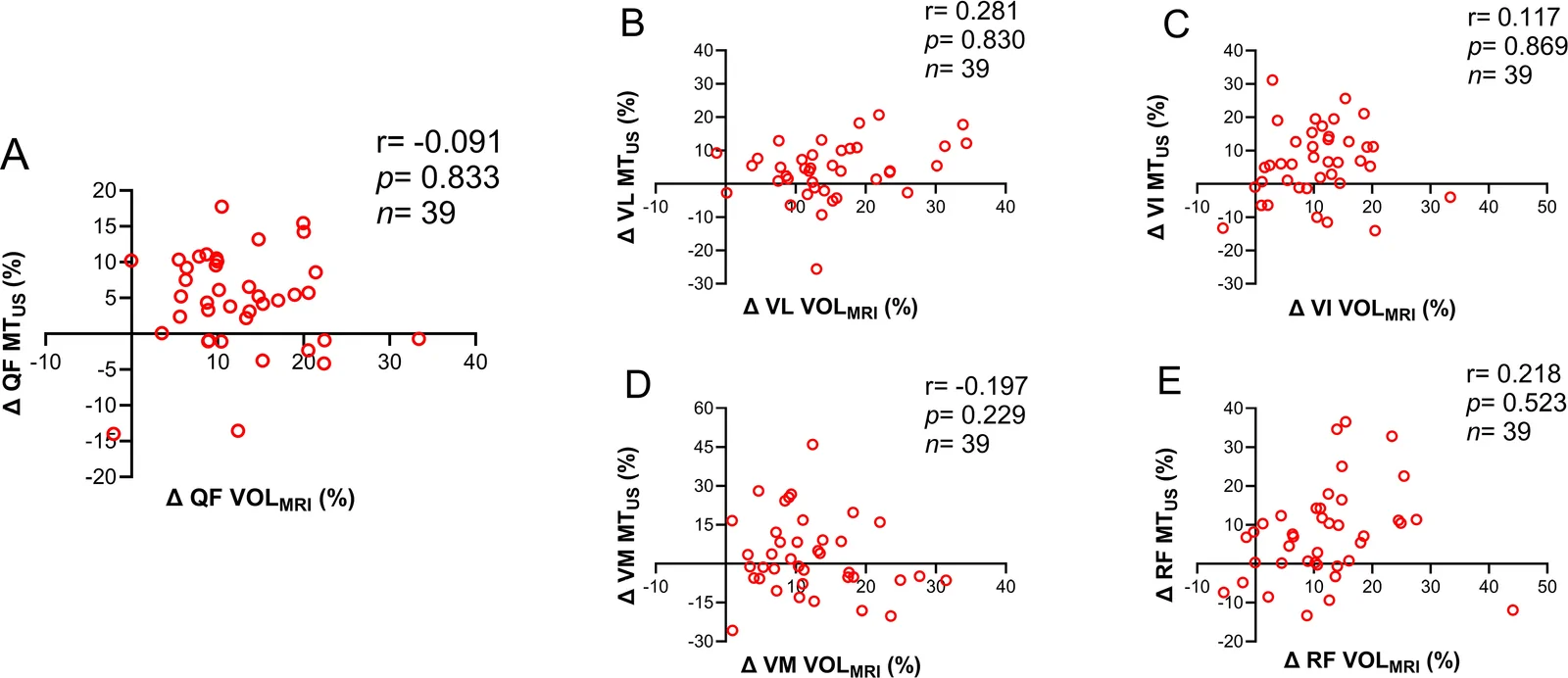
Bivariate correlations between percentage changes (Δ) in MT_US_ and changes in VOL_MRI_ of the overall quadriceps femoris (QF; A) and the constituent muscles (B–E) following 15 weeks of lower-body resistance training. r values derived from Pearson’s product-moment correlations are displayed. Adjusted *P* values are also displayed.

### Pre-Training Cross-Sectional Bivariate Correlations Between MT_US_ and MRI Measures of Muscle Size

To provide context for the relationship of RT-induced changes between measures of muscle size, the pretraining relationships between MT_US_ with both ACSA_MRI_ and VOL_MRI_ were also assessed. At pretraining, MT_US_ showed weak to strong relationships with ACSA_MRI_ for the overall QF (r = 0.525, adjusted *P* = 0.002), and for VI, VL, RF (0.426 ≤ r ≤ 0.712, 0.001 ≤ adjusted *P* ≤ 0.009), but not the VM (r = 0.103, adjusted *P* = 0.591). There were also weak to moderate pretraining relationships between ultrasound MT_US_ and VOL_MRI_ for the overall QF (r = 0.455, adjusted *P* = 0.005) and for VI, VL, RF (0.459 ≤ r ≤ 0.670, 0.001 ≤ adjusted *P* ≤ 0.005), but not the VM (r = 0.030, adjusted *P* = 0.856).

## DISCUSSION

This study investigated whether group-level ΔMT_US_ of the QF, and its constituent muscles, were comparable to changes in MRI-based measures of muscle size and if individual ΔMT_US_ was associated with corresponding ΔACSA_MRI_ and ΔVOL_MRI_ following RT. ΔMT_US_ significantly underestimated increases in ΔACSA_MRI_ and ΔVOL_MRI_ for the overall QF and two out of four constituent muscles (VL and VM), with a mean offset/underestimate of more than half the change observed with criterion MRI measures, and an average error that was typically more than the change in the criterion MRI measures. Furthermore, there were no relationships between individual ∆MT_US_ and ∆ACSA_MRI_ or ∆VOL_MRI_ (expressed in absolute or relative terms) for the overall QF or for any constituent muscle.

Various imaging methods are used by researchers and practitioners to assess changes in muscle size following RT and other interventions. MRI, the current criterion imaging modality for the measurement of muscle size and changes in muscle size, is expensive and not widely accessible, whereas measurements of MT_US_ are widely accessible. However, there has been a paucity of research examining the validity of ΔMT_US_ as an index of muscle growth after RT, compared with criterion measurements such as ΔACSA_MRI_ and ΔVOL_MRI_. The current study involved a relatively large cohort of 39 participants completing a prolonged 15-week RT intervention that led to a relatively large hypertrophic response (QF ΔVOL_MRI_ +12.4%). Considering the muscle growth induced by RT, MT_US_ was able to detect group-level pre-to-post changes in three of four constituent muscles and the overall QF. However, ∆MT_US_ significantly underestimated changes in MRI measures for the overall QF, the VM, and importantly the VL; a muscle solely measured within some RT interventions (25,47–50) and often assumed to represent skeletal muscle hypertrophy of the whole QF (16). The observed underestimations corresponded to ∆MT_US_ capturing less than half (overall QF), and less than one-third (VM and VL), of the change in the criterion MRI measures (ΔACSA_MRI_ or ΔVOL_MRI_) for these muscles. However, for VI and RF, ΔMT_US_ did not statistically differ compared with corresponding changes assessed with MRI. The reason for the contrasting group-level results for the VM and VL compared with the RF and VI is currently unclear. Overall, these results indicate ΔMT_US_ markedly underrepresents RT-induced muscle hypertrophy for the overall QF and two individual muscles compared with criterion MRI measures. These results are partly in alignment with a prior small sample (*n* = 10), short-duration (6 weeks of RT) study where ΔMT_US_ of the elbow flexors did not consistently equate to ΔACSA_MRI_ (32). The underestimation by ∆MT_US_ did not appear to be due to poor measurement reliability, as all three measures of muscle size (MT_US_, ACSA_MRI_, and VOL_MRI_) were found to be highly reliable with a mean within-participant between-session CV of <2%. The underestimation by ∆MT_US_ compared with criterion measures could be due to differences in the imaging modality (e.g., ultrasound vs MRI) or geometry between the measurements. From a geometric perspective, considering the uniform growth of cylindrical muscles, the ΔMT is expected to increase in proportion with the linear dimension (radius), whereas ΔACSA would be expected to increase to the square of the linear dimension (i.e., radius^2^). While the quadriceps muscles clearly have much more complex three-dimensional shapes than simple cylinders (e.g., the crescent-shaped cross-section of the VL and VI at mid-thigh; see Figure 1 B, Supplemental Digital Content, available at https://links.lww.com/MSS/D445), we found that ΔMT_US_ did markedly underestimate ΔACSA_MRI_ and ΔVOL_MRI_, which may in part be due to the geometric difference between MT_US_ compared with measures of ΔACSA_MRI_ and ΔVOL_MRI_. A greater understanding of the geometric nature of RT induced muscle growth by future investigations would likely inform the validity of different measures of muscle growth.

Considering individual participant data, the present investigation found no relationships between absolute or relative ΔMT_US_ and corresponding MRI measures (ΔACSA_MRI_ or ΔVOL_MRI_) for the overall QF or its constituent muscles. This was despite the pretraining cross-sectional analysis revealing significant associations between MT_US_ and MRI measures (ACSA_MRI_ or VOL_MRI_) of QF size, in accordance with previous findings (17–23). In addition, after RT, the average individual error for ∆MT_US_ compared with criterion MRI measures was large and typically more than the mean change assessed by the criterion measures. Thus, while MT_US_ appears to be related to measures of muscle size per se, it appears to have insufficient precision to assess the changes in muscle size after RT. We are not aware of any previous investigations documenting the error of ∆MT_US_ measurements; however, previous studies with small samples for the assessment of relationship (*n* ≤ 12) have presented somewhat mixed findings, with ΔMT_US_ reported to be unrelated (33) and related (20) to ΔACSA_MRI_, and unrelated to ΔVOL_MRI_ (20). The current study involved a much larger sample (*n* = 39) and substantially greater hypertrophy (VL ΔACSA_MRI_ 14.4% vs 5.2%) than the only report of a positive relationship of ΔMT_US_ and an MRI measure of hypertrophy (20), presumably due to the longer RT duration (15 vs 12 weeks) and number of quadriceps exercises within the current study. Furthermore, a recent study also found no relationship between ΔMT_US_ and either ΔACSA_MRI_ or ΔVOL_MRI_ after disuse atrophy (51), suggesting that the limitations of MT_US_ to assess muscle size changes applies to both atrophy and hypertrophy. Therefore, the preponderance of evidence suggests that ΔMT_US_ are not associated with criterion MRI measures of hypertrophy/atrophy, likely because of the high error of individual change data, and thus ΔMT_US_ does not predict RT-induced muscle growth observed with criterion multidimensional MRI muscle size measures.

Overall, ΔMT_US_, a one-dimensional measure of muscle hypertrophy, does not appear to be an accurate or valid index of RT-induced muscle growth. Consequently, caution should be taken when using or interpreting findings generated with MT_US_. For example, the current results demonstrate that ΔMT_US_ is not interchangeable with criterion measures (ΔACSA_MRI_ and ΔVOL_MRI_) and thus reviews/meta-analyses should not consider these measures as equivalent or similar. The apparent lack of accuracy or association with criterion measures of muscle growth exhibited by ΔMT_US_ could be because muscle growth is a three-dimensional phenomenon involving changes in width, and potentially also aspects of length (e.g., fiber or fascicle length), as well as thickness (2,16,52). Alternatively, MT_US_ measures of muscle size may simply lack the resolution for accurate measurements of muscle growth. Although it is noteworthy that ultrasound measurements of ACSA have been found to generate group mean changes (53) that are similar to, and individual changes that are strongly correlated (r = 0.85; R^2^ = 72% (54)) with, ΔACSA_MRI_ of the VL after RT. This evidence suggests that the poor validity of ΔMT_US_ may be primarily due to its one-dimensional nature rather than the imaging modality per se. Consequently, the use of ultrasound ACSA appears preferable to MT_US_ for assessing skeletal muscle hypertrophy, and the development of panoramic ultrasound, often featuring automatic image construction, now makes the possibility of attaining ACSA measures from large lower-body muscles more accessible.

This study was not without its limitations. The idea for this study was to collect the different muscle size measurements in the way they are conventionally used (i.e., independently), while collecting ΔMT_US_ at multiple locations per muscle, and with ΔACSA_MRI_ and ΔMT_US_ measurements at similar anatomical locations, at and around the location of maximum ACSA, respectively. However, this approach meant that the anatomical locations of ΔMT_US_ and ΔACSA_MRI_ were not completely identical, which may possibly have contributed to their lack of similarity. Additionally, it may also have been preferable to assess MT_US_ and MRI measures in an identical posture. Theoretically, a more flexed knee joint angle (i.e., as used for ultrasound vs MRI assessments in the current study) could be expected to produce smaller MT_US_ measures due to decreased overlap between myosin and actin contractile filaments. However, manipulation of joint angle has previously been shown to not affect MT_US_ measures (55). The main focus of the current study was the change from pre to post, which was done with a consistent protocol and posture within each measure, and thus it is unclear how any difference in posture may have affected the change measurements. Finally, although the results of the current study are directly applicable to RT-induced hypertrophy of the QF within a young healthy adult sample, these findings require replication within other populations and different muscle groups.

## CONCLUSIONS

In conclusion, group-level ΔMT_US_ for the overall QF and multiple constituent muscles markedly underestimated RT-induced muscle hypertrophy assessed using criterion MRI measures, and ΔMT_US_ were unrelated to MRI-derived individual changes in muscle size (ΔACSA_MRI_ or ΔVOL_MRI_). Consequently, caution should be taken when considering the use of MT_US_ or interpreting findings generated with this measure within the context of RT-induced muscle growth.

The authors would like to thank Jim Muddimer and Mike Myatt for their technical expertise, and radiographer Julie Thompson.

This study was funded in part by the Collagen Research Institute (CRI), Kiel, Germany. No conflicts of interest were disclosed.

The results of the study are presented clearly, honestly, and without fabrication, falsification, or inappropriate data manipulation. The results of this study do not constitute endorsement by the American College of Sports Medicine. MPF is supported by the National Institute for Health Research (NIHR) Applied Research Collaboration East Midlands (ARC EM).

The views expressed are those of the author and not necessarily those of the NIHR, NHS or the Department of Health and Social Care.

## Supplementary Material
