# Supplementary material for: Ultrasound Muscle Thickness Is a Poor Index of Criterion Magnetic Resonance Imaging Measures of Resistance Training-Induced Muscle Growth
Source: Med Sci Sports Exerc. 2026 Jul 7;58(11):2466–76. doi: 10.1249/MSS.0000000000004078 (PMC13652124; doi:10.1249/MSS.0000000000004078)
Supplement: Supplementary file 1 [file msse-58-2466-s001.pdf]

SUPPLEMENTARY MATERIAL

Supplementary material 1. Example images of the quadriceps femoris and its constituent muscles for: (A) ultrasound imaging; and (B) axial magnetic resonance imaging (MRI). Ultrasound muscle thickness was measured at two to three locations within each ultrasound image to closely equate the location along the femur where the peak of the anatomical cross-sectional area-femur length curve occurs for each muscle based on prior MRI studies (1, 44). Ultrasound image locations: VL, vastus lateralis and VI, vastus intermedius (image centred on 50% of femur length); RF, rectus femoris (image centred on 75% of femur length); and VM, vastus medialis (image centred on 20% of femur length; 0% is knee joint space).

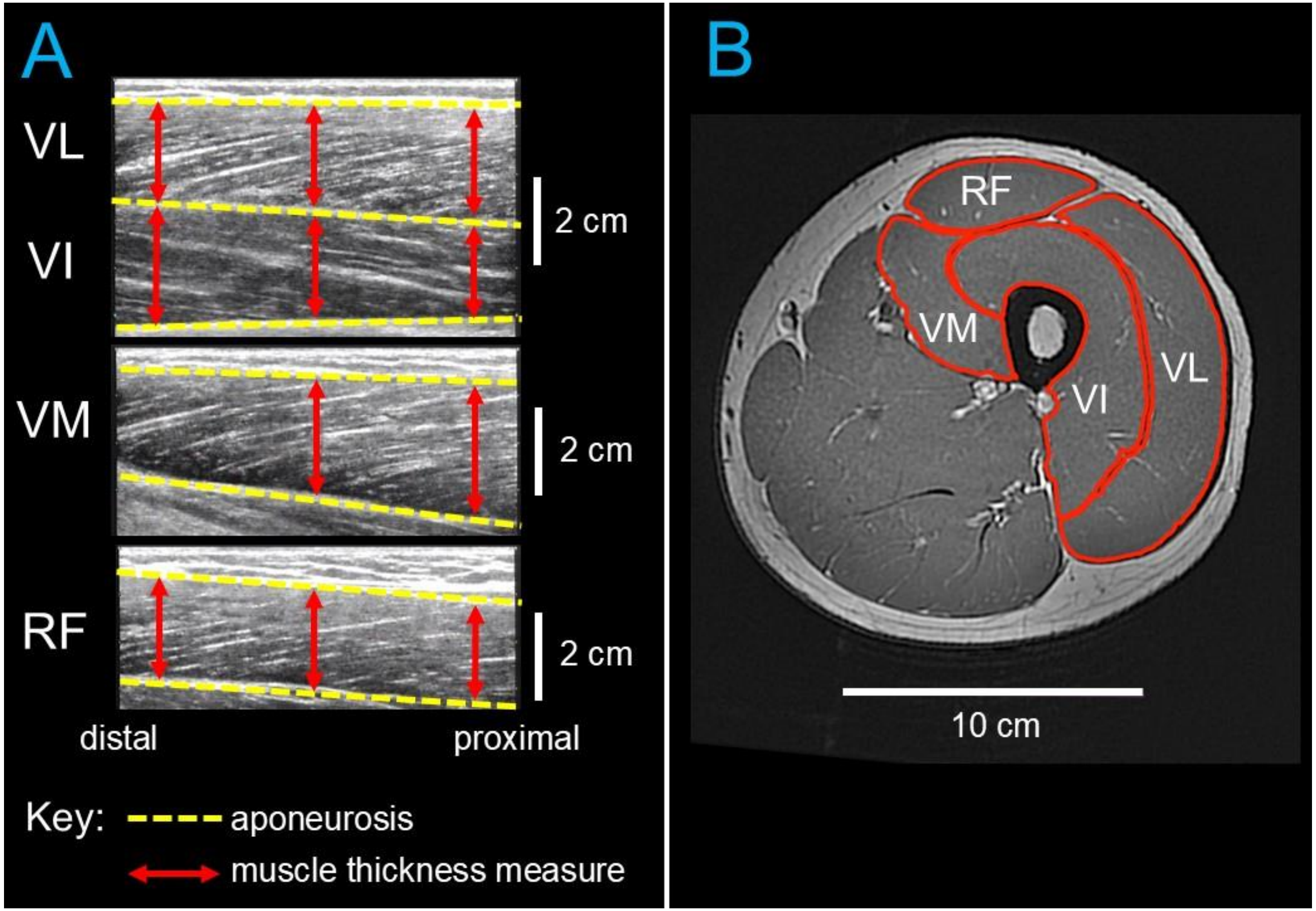

Supplementary material 2. Bivariate correlations between (A) absolute and (B) percentage changes (Δ) in ultrasound muscle thickness (MT_{US}) and magnetic resonance imaging (MRI) measures of muscle size (maximum anatomical cross-sectional area [$ACSA_{MRI}$] and volume [VOL_{MRI}]) following 15 weeks of lower-body resistance training (*n*=39).

	r	adjusted *p*	r value descriptor
**(A)**			
Δ MT_{US} (mm) vs. Δ $ACSA_{MRI}$ (cm^2):			
VL	0.236	0.490	"very weak"
VI	0.240	0.564	"very weak"
VM	-0.096	0.745	"very weak"
RF	0.198	0.565	"very weak"
QF	-0.110	0.634	"very weak"
Δ MTUS (mm) vs. Δ VOL_{MRI} (cm^3):			
VL	0.170	0.669	"very weak"
VI	0.129	0.866	"very weak"
VM	-0.153	0.371	"very weak"
RF	0.422	0.140	"weak"
QF	-0.015	1.431	"very weak"
**(B)**			
Δ MT_{US} (%) vs. Δ $ACSA_{MRI}$ (%):			
VL	0.281	0.830	"very weak"
VI	0.242	0.685	"very weak"
VM	-0.117	0.564	"very weak"
RF	0.086	1.002	"very weak"
QF	-0.152	0.393	"very weak"
Δ MTUS (%) vs. Δ VOL_{MRI} (%):			
VL	0.281	0.830	"very weak"
VI	0.117	0.869	"very weak"
VM	-0.197	0.229	"very weak"
RF	0.218	0.523	"very weak"
QF	-0.091	0.833	"very weak"

p values were derived from Pearson's product moment correlations. Adjusted *p* values were derived as part of the Benjamini-Hochberg procedure as follows: *p* x (m/i), where m is the total number of statistical tests (*n*=20) and *i* is the *p* value rank. None of the adjusted *p* values were significant (i.e. $p<0.05$). VL, vastus lateralis. VI, vastus intermedius, VM, vastus medialis. RF, rectus femoris. QF, quadriceps femoris. QF measures were calculated by summing the values of the 4 constituent muscles of the QF.
